# Editorial: Host Innate Immune Response and Its Impact on Pulmonary Pathogenesis During Influenza Virus Infection

**DOI:** 10.3389/fcimb.2021.779411

**Published:** 2021-11-09

**Authors:** Yee-Joo Tan, Victor C. Huber, Eugenio D. Hottz

**Affiliations:** ^1^ Infectious Diseases Translational Research Programme and Department of Microbiology and Immunology, Yong Loo Lin School of Medicine, National University of Singapore, Singapore, Singapore; ^2^ Division of Basic Biomedical Sciences, Sanford School of Medicine, University of South Dakota, Vermillion, SD, United States; ^3^ Laboratory of Immunothrombosis, Department of Biochemistry, Federal University of Juiz de Fora (UFJF), Juiz de Fora, Brazil

**Keywords:** influenza virus, immunopathogenesis, inflammation, host-pathogen interaction, COVID-19

In this Research Topic issue on host innate immune responses during Influenza virus infection, we compiled 2 original articles and 4 reviews providing timely insights into influenza pathogenesis. The two original articles in this Research Topic investigate receptors and factors controlling viral replication by using gene silencing strategies to dissect innate immunity and anti-viral responses against influenza. The review articles in this Research Topic highlight major roles of innate and adaptive immune cells, especially mast cell, natural killer (NK)-cell and T-cell responses during infection, and present parallels between influenza and coronavirus disease 2019 (COVID-19), exploring similarities and differences regarding pulmonary and extrapulmonary complications.

The complex network of viral-host interactions determines the replication efficiency of a virus and its pathogenicity. Two articles under this Research Topic revealed host factors influencing influenza A virus (IAV) replication, the chemokine CCL5 that inhibited replication by up-regulating the restriction factor SAMHD1; and the cytosolic sensor NLRC5, which served as a pro-viral factor. Silva et al. determined if ligands of the CCR5 receptor could enhance expression of restriction factors. Interestingly, treatment of the A549 human pneumocyte cell lineage with CCR5 agonists, namely CCL3, 4 or 5, prior to infection with influenza A virus reduced IAV progeny. These ligands increased mRNA levels of SAMHD1 and siRNA knockdown of SAMHD1 increased viral replication, implying that SAMHD1 mediates CCR5-induced restriction. Besides inhibiting viral replication, CCL5 also reduced virus-induced cell death in a SAMHD1-dependent manner. This study demonstrates that the up-regulation of β-chemokines, which bind to CCR5 to attract inflammatory cells to the site of infection, can also up-regulate SAMHD1 to reduce viral replication. Thus, further studies can be directed at identifying signaling modulation that can induce viral restriction factors capable of controlling infection effectively. Chothe et al. studied the regulation of NLRC5 in chicken cells during infection by either low or high pathogenicity avian influenza A viruses. Transcriptome analysis of primary chicken lung cells revealed that NLRC5 was up-regulated by infection with both strains of avian IAV. Infection of the chicken macrophage MQ-NCSU cell line confirmed the up-regulation of NLRC5 at both mRNA and protein levels during IAV infection. Knockdown of NLRC5 with siRNA decreased viral replication, implying that NLRC5 is a pro-viral factor. In addition, transcriptional analysis of NLRC5 knock-down cells showed that reduced NLRC5 modulated the NFκB signaling pathway, enhanced viral defense and decreased innate cytokine production in infected macrophages, indicating that NLRC5 plays a role in enhancing innate immunity during infection. This study suggests NLRC5 as a potential host-targeting factor for future research in influenza treatment, which has a higher barrier to drug resistance than drugs targeting the virus.

Two review articles from Dr. Silke Paust’s group highlight the participation of NK, T, and mast cells in injurious and protective immune mechanisms during influenza infection, bringing attention to the essential balance between resistance and tolerance to infection outcomes. Murphy-Schafer and Paust discuss the dual role of mast cells in pulmonary pathogenesis, playing major roles in inflammatory amplification through cytokine, amine, and protease release, but also orchestrating the resolution of inflammation at the mucosal site. This review surmises the main pathways of mast cell recruitment and activation during influenza through cytokine, chemokine, Fc, and pattern recognition receptor engagement, and propose a potential contribution of mast cells in resolution of inflammation during influenza through IL-6 and IL10 signaling. Franck and Paust provide an extensive review on the main NK and T cell phenotypes and effector functions at homeostasis and in lung pathology during influenza infection. The review points out key roles of NK and T cells in inflammatory network regulation and cytotoxic-mediated viral clearance. The authors compile reports on the main mechanisms of cellular activation and pathogen evasion, and provide a debate on the major role of these cells in host defense and pathology, as well as their applicability as therapeutic targets against influenza infection.

While putting together this Research Topic, the world was faced with the first coronavirus pandemic on record. The virus causing this pandemic is the severe acute respiratory syndrome coronavirus 2 (SARS-CoV-2). Since its emergence in late 2019, members of the scientific community have been applying knowledge in their respective fields toward solving the COVID-19 pandemic problem. In particular, labs that study influenza began to draw parallels between host-pathogen interactions that direct severe influenza pneumonia and those leading to deadly COVID-19. In this special issue, we present two timely review articles that add to the literature in this area. The review written by Kevan Hartshorn details the contribution of innate immunity toward the development of severe influenza infections, with emphasis on the damage that occurs due to induction of strong inflammatory responses. This review then discusses the therapeutic potential for innate inhibitors, like surfactant protein D and antimicrobial peptides, to limit severe lung infections at early stages of host-pathogen interaction. These responses are discussed in the context of our current knowledge on COVID-19, which helps to direct these findings in the influenza research field toward this new viral threat. Similarly, the review by Fernanda Andrade et al. explores our knowledge of influenza virus persistence and pathological consequences of the immune response in obese individuals. Since obesity can have a dramatic effect on the outcomes of respiratory infections, our past knowledge of influenza infections in this at-risk population is directing what we are learning about SARS-CoV-2. Specifically, the impact of obesity on host immune responses and cardiovascular disease are highlighted in this review. Together, these two articles demonstrate common links between severe influenza and COVID-19, with emphasis on innate immune responses and obesity as a risk factor. These reviews surmise the many advances made in our understanding of host-pathogen interactions, which allows us to inquire if common therapeutic approaches can be applied to limit severe complications associated with either of these viral pathogens.

Overall, this Research Topic expands our understanding of immune mechanisms of severe influenza pneumonia and provides further insights on pathological processes of pulmonary viral infections.

## Author Contributions

All authors listed have made a substantial, direct and intellectual contribution to the work, and approved it for publication.

## Conflict of Interest

The authors declare that the research was conducted in the absence of any commercial or financial relationships that could be construed as a potential conflict of interest.

## Publisher’s Note

All claims expressed in this article are solely those of the authors and do not necessarily represent those of their affiliated organizations, or those of the publisher, the editors and the reviewers. Any product that may be evaluated in this article, or claim that may be made by its manufacturer, is not guaranteed or endorsed by the publisher.

